# Nozzle Shape Guided Filler Orientation in 3D Printed Photo-curable Nanocomposites

**DOI:** 10.1038/s41598-018-22107-0

**Published:** 2018-02-28

**Authors:** Taeil Kim, Ramita Trangkanukulkij, Woo Soo Kim

**Affiliations:** Additive Manufacturing Laboratory, School of Mechatronic Systems Engineering, Simon Fraser University, BC, Canada

## Abstract

Here, we report guided orientation of silver nanowires (AgNWs) in extruded patterns with photo-curable 3D printing technology. A printable conductive composite material composed of polymer matrix and silver nanowires shows significantly varied electrical properties depending on the cross-sectional shape of printing nozzles: flat or circular. The composite is designed to have highly conductive AgNWs and a dielectric polymer matrix like photo-curable methacrylate resin. The dielectric permittivity of photo-curable composite resin with 1.6 vol. % of AgNWs printed through a circular nozzle showed 27. However, the same resin showed much lower permittivity with 20 when it is printed with a flat nozzle. The cross-sectional sample morphology shows that AgNWs printed with a circular nozzle are aligned, and AgNWs printed with a flat nozzle are randomly distributed. A computational simulation of paste extrusion with two different nozzle shapes showed clearly different fluidic velocities at the nozzle exit, which contributes to different fiber orientation in printed samples. A radio frequency identification sensor is fabricated with 3D printed composite using a flat nozzle for the demonstration of AgNW based 3D printed conductor.

## Introduction

Polymer nanocomposites (PNCs) became a promising solution of various applications for their flexibility and processability as well as differentiated mechanical, electrical properties compared with pristine polymers^1^. Recent development of PNCs demonstrates functional properties as well as easy processibility required in current electronic device industry. Especially materials with high conductivity or high dielectric permittivity gained great attention. Materials with high conductivity can be applied to fabricate basic electrical parts such as interconnections, inductors, and electrodes of batteries^2^. PNCs with high conductivity can be utilized for 3D printing of electromagnetic interference (EMI) shielding applications which will contribute to miniaturization of portable electronic devices. Materials with high dielectric permittivity can be applied to parts with high capacitance and improved energy storages^3^. PNCs with high dielectric permittivity can be applied to flexible and transparent parts of wearable electronic devices^4–6^.

Various types of PNCs were investigated to prepare a flexible material with high conductivity or high dielectric permittivity^6–8^. Numerous kinds of different polymers such as epoxy, Polyimides (PI), Poly (methyl methacrylate) (PMMA), and Polydimethylsiloxane (PDMS) were used as a matrix in PNCs. Ferroelectric ceramic fillers such as BaTiO_3_, PbTiO_3_, and TiO_2_ or conductive nano wires such as carbon nanotube (CNT) and AgNW were applied to attain enhanced electrical properties^8–11^. But high loads of ceramic fillers, which is required for high dielectric constant, sometimes worsen the mechanical property of the material^12^. PNCs with conductive fillers may exhibit better mechanical flexibility as well as improved electrical properties with lower volume fraction of fillers compared to PNCs with ceramic fillers. A PNC with conductive fillers like AgNWs can be conductive if these conductive nanoscale fillers form a conductive path called as a percolated network which enables the electrons travel through the material. Electrons can move from one conductive filler to another if they are in contact or jump between conductive fillers if they are close to each other, known as tunneling effect^13^. On the other hand, conductive nanoscale fillers and polymer matrix may be arranged in an ordered structure so that the conductive nanoscale fillers can work as pairs of electrodes of capacitors. And the polymer matrix between nanoscale fillers can work as dielectric to form millions of nanoscale capacitors and exhibit high dielectric properties^11^.

In this study, we chose AgNW as a nanoscale conductive filler to prepare PNCs in order to utilize the high anisotropic character of AgNWs. AgNW is a rod-like anisotropic structure. So its properties may be controlled more effectively by alignment in a matrix material compared to other isotropic nanoscale fillers with a spherical structure. Also, high aspect ratio (100~500) of AgNW induces lower percolation threshold compared to spherical fillers^14^. Therefore, AgNW has higher conductivity compared to other fillers like carbon nanotube for highly conductive or highly dielectric applications^2,15^. Also, the large surface area of AgNW contributes to the efficient improvement of conductive or dielectric property of polymers.

The orientation of fillers in the matrix of nanocomposites affects conductive or dielectric properties as well as mechanical properties. Research has been focused on enhanced mechanical property of nanocomposites with aligned distribution of fillers^16,17^. The relation between electrical properties such as conductivity or permittivity and the orientation of fillers has been also investigated^19,20^. If it is possible to use the same compositional materials with the same volume fraction of AgNWs to differentiate electrical properties, then it makes price competitiveness. And if it is possible to control the alignment and orientation of filler distribution, then composites’ electrical properties can be tuned by a small part of manufacturing process. For example, changing the nozzle shape of an extruder can give more efficient processability. In this report, it is demonstrated that the electrical properties of composite materials can be tunable through nozzle dependent extrusion 3D printing.

Controlling the filler orientation of PNCs may improve electrical properties. One of the simple and useful techniques to control filler orientation in extrusion printing is to change nozzles’ shapes at the end of the extruder. In an extrusion process through nozzles, the converging parts of nozzles will increase and the diverging parts of nozzles will decrease the level of alignment of fillers along the extruded direction^21^. By applying these converging and diverging nozzles in extrusion printing, we can study the relation between the orientation of nanoscale fillers and composites’ electrical properties. It is known that the fabrication process determines material’s properties. There are many 3D printing methodologies such as solution casting, filament printing, extrusion printing, and Digital Light Processing (DLP)^11,18^. The extrusion printing is an adequate printing method for nanocomposite materials because it enables us to control filler orientation more easily than other fabrication methods such as solution casting and DLP. This study mainly demonstrates attaining different electrical properties with the nanocomposite, which has same concentration of AgNWs in polymer composites by controlling the orientation of fillers.

A silicone rubber (Ecoflex® 00–30) was utilized as a polymer matrix in a PNC with AgNWs. This PNC was extruded and dried at room temperature. Initially distributed AgNWs inside of polymer sank down to the bottom region of printed sample during the drying process. Because AgNWs are heavier than silicone polymer, they are able to move to the bottom of prints. Therefore, more AgNWs were precipitated at the bottom of a printed sample in case of a PNC with a higher volume fraction of AgNWs. In order to solve this sinking issue, Print & Cure (P&C) system was newly developed to fix AgNWs’ distribution inside of polymer matrix using by UV light right after the extrusion. It has been reported with direct writing of carbon nanotube/polymer nanocomposite materials^22,23^. In P&C system, extruded AgNWs were not sunk down and stayed in the layer where they were printed because methacrylate polymer resin is photo-cured right after printing. Eventually, the cured composite holds AgNWs as printed. Photo-curing is an appropriate technique to keep distribution of AgNWs as printed. The P&C resin, a UV curable resin composed of oligomer (Urethane triacrylate), photo initiator (Phenylbis phosphine oxide), and monomer (Methacrylic acid) is prepared as a matrix which will be cured by UV light with 405 nm in wavelength. The change depending on the extent of alignment of AgNWs was investigated.

**Figure 1:**
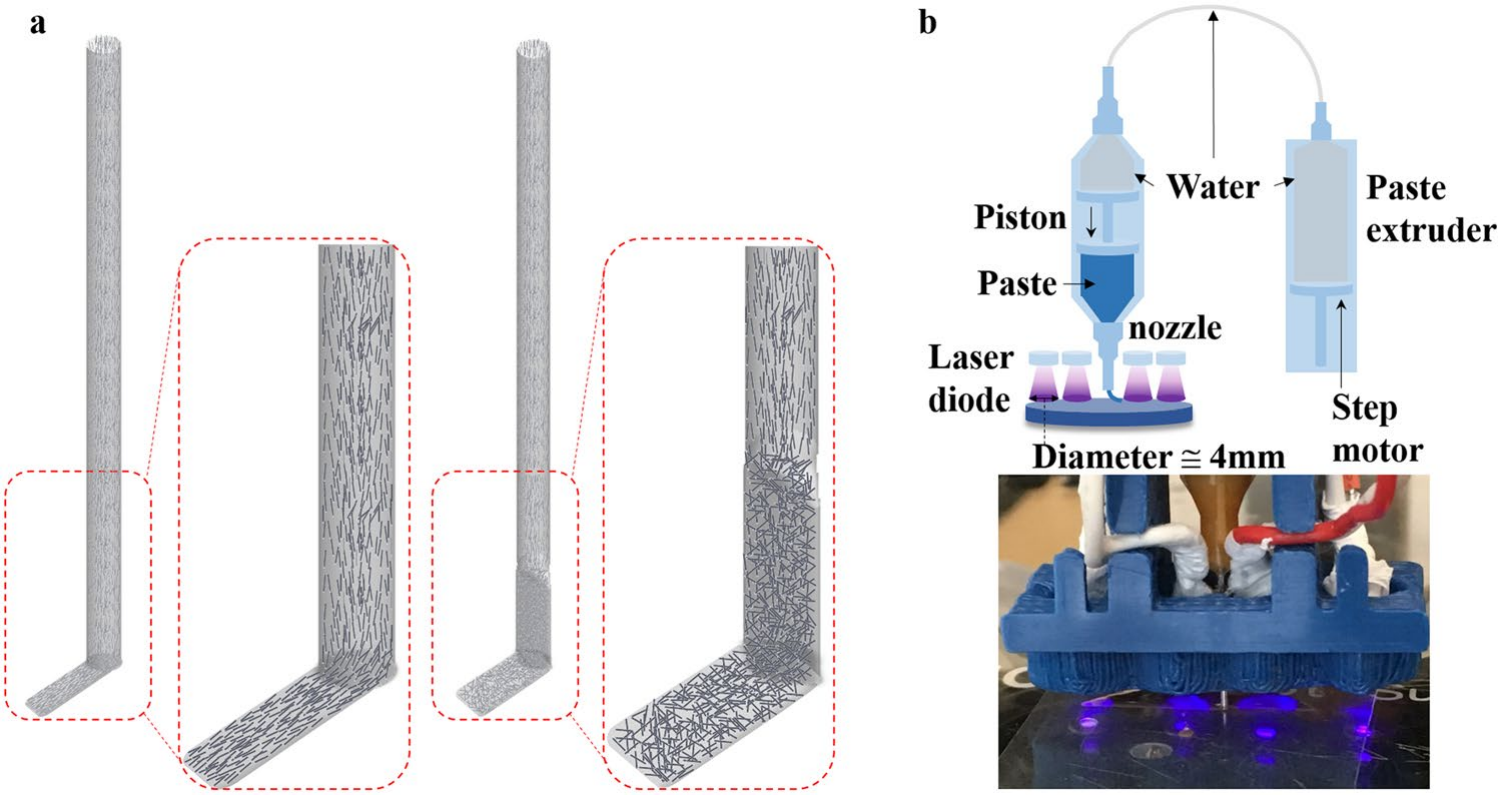
Schematics of the extrusion at nozzles and Print & Cure extrusion 3D printing system. (**a**) The scheme of print-induced aligned/random distribution of high aspect ratio fillers of a circular nozzle and a flat nozzle, (**b**) The scheme of Print & Cure extrusion 3D printing system (up) and a close-up image of Print & Cure printing head (bottom).

Figure 1a shows a schematic of the extrusion of high aspect ratio fillers in a matrix and print-induced distribution of fillers with two kinds of nozzle tips. The left image describes the extrusion from a circular nozzle which has a cylinder shape with a circular cross section. The right image shows the extrusion from a flat nozzle which has a cylinder shape with an ellipse-like flat cross section in the lower part at the end of nozzle. These two images also visualize the assumption that the circular nozzle generates aligned orientation of fillers in a printed sample, and the flat nozzle generates random distribution of fillers after extrusion. This assumption was investigated and verified by experiments as well as a computational simulation. The actual circular nozzle has a diameter of 840 µm and length of 17 mm. The flat nozzle has the same length as the circular nozzle with tapered end part of the nozzle: 3 mm. The tapered flat part at the end of the nozzle has an ellipsoidal cross section with the wider and the narrower inner diameters of 1.2 mm and 360 µm respectively.

Figure 1b shows the scheme of P&C extrusion 3D printing system and the real image of its printing head. Four laser diodes give UV light with the wavelength of 405 nm to the printed object and cure it. The focal range of UV light from one diode is about 4 mm in diameter. These diodes are arranged on a line which is parallel to the printing direction along X axis for the most effective curing. It is important to keep diodes as close as possible from the printing surface in order to secure best curing performance because the intensity of the light is dramatically decreased when the distance between diodes and the printing surface is increased.

## Results and Discussion

**Figure 2:**
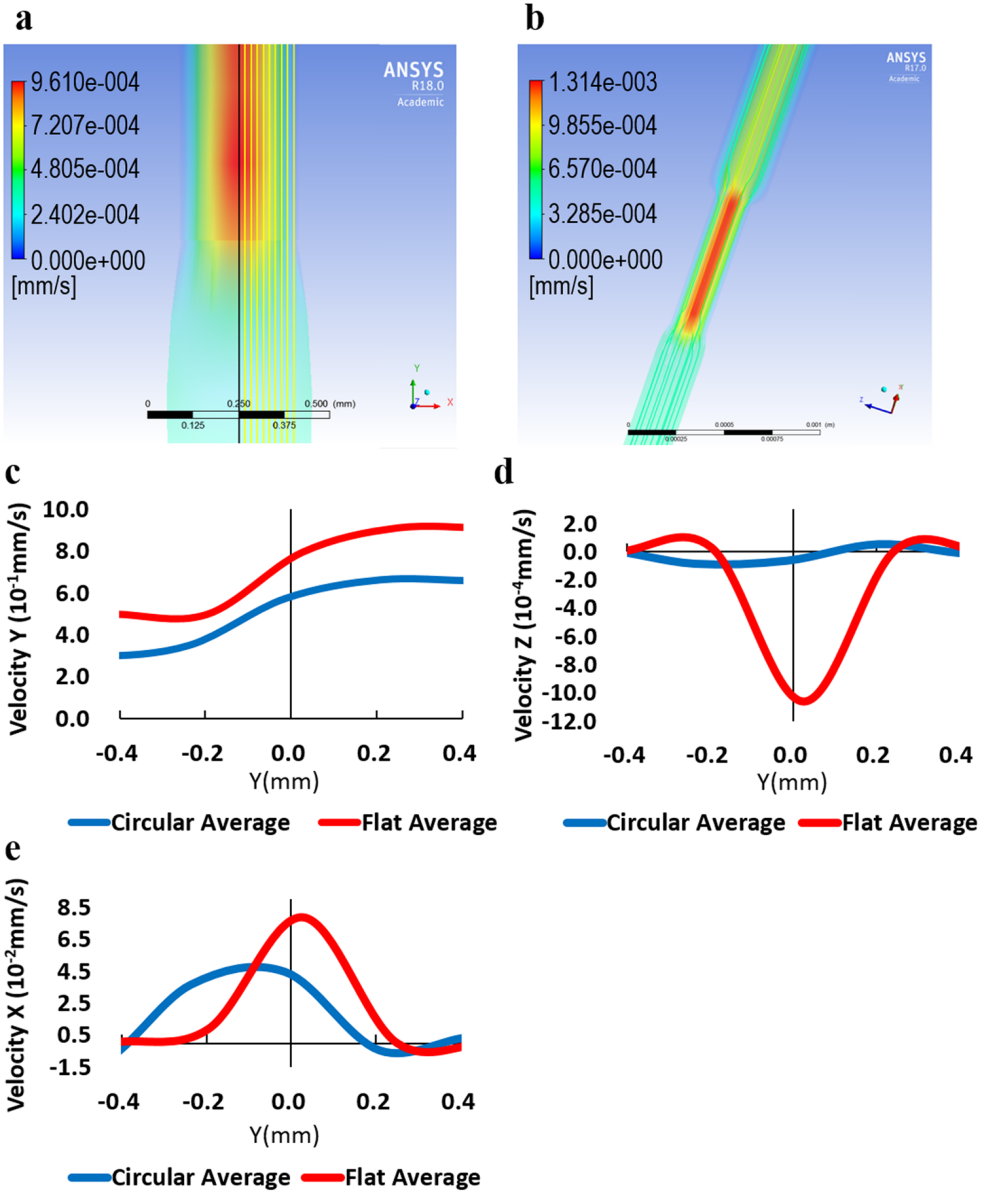
Filler extrusion simulation depending on nozzle shapes. Velocity profile of the fluid extruded at a circular nozzle **(a)** and a flat nozzle **(b)**, and Average Velocity Y, Z, and X **(c**, **d**, and **e)** of fluid around the exit of a circular nozzle and a flat nozzle.

In this section, experimental sample morphology is investigated with velocity profile images to prove AgNWs’ nozzle dependent extruding distribution in PNCs, then measured dielectric permittivity of printed samples with two types of nozzles are discussed. Finally, randomly extruded AgNWs’ conductive printing was demonstrated for the application of RFID tag. The numerical model was solved by ANSYS polyflow using Cross law as the governing equation. The geometry of computational domain was defined and generated as a model composed of two parts which are the fluid in a nozzle and the extrudate for two types of nozzles respectively. Figure 2a,b show the simulation result of extrusion printing with a circular and a flat nozzle. This simulation was designed as a decoupled model^24^. It is assumed that the nanoscale fillers will orient following the flow of polymer matrix because the volume fraction of nanoscale fillers is very low. The extrusion direction is from positive Y to negative Y along the Y axis. A type of generalized Newtonian fluid, Cross model was used for the calculation of simulation.

1 $${\rm{\eta}}=\frac{{\rm{\eta}}_{0}}{1+{(\lambda \gamma)}^{m}}\,$$

where, ɳ_0_ = zero-shear-rate viscosity, λ = natural time (inverse of the shear rate at which the fluid changes from Newtonian to power-law behavior), ɤ = shear rate, and m = power-law index. Cross model exhibits shear-thinning behavior, which is dominant viscoelastic behavior of majority of polymers. The degree of dependence of viscosity on shear rate in shear-thinning region can be easily adjusted by changing power-law index. For example, lower power-law index means less dependence of viscosity on shear rate. Zero-shear-rate viscosity was estimated as 1500 cps, because the P&C resin with 1.6 vol. % of AgNWs shows viscosity around 1500 cps. Natural time was set as 0.2 s, which is close to the Newtonian limit where natural time is 0. Though Cross initially proposed that power-law index = 2/3 was satisfactory for large number of cases, power-law index of this study was set as 0.3 to reflect the lower dependence of viscosity on shear rate of the AgNW P&C resin composite of this study^25^. As it is assumed that there is a uniform velocity profile at the end of extrudate, normal and tangential forces were set to zero.

Since the problem involves a free surface, the domain was divided into two subdomains. Subdomain 1 is the fluid inside of a nozzle. Subdomain 2 is the extrudate outside of a nozzle. Boundaries were defined only at the inlet, nozzle wall, free surface, and outlet (at the end of extrudate), No boundary was defined at the nozzle exit where the fluid in the nozzle interfaces the fluid in the extrudate. Thus, we didn’t have to justify the inaccuracy at the boundary at the nozzle exit. Boundary conditions are as following. At the inlet, volumetric flow rate Q is set as 4 × 10^−5^ cm^3^/s. The boundary between the nozzle wall and the fluid is set as zero velocity because the fluid is assumed to stick to the wall, which is the no-slip condition. Hence the fluid has no velocity relative to the nozzle wall at this solid-fluid interface. At the boundary of the free surface after nozzle exit, velocity profile is not uniform. The velocity field must be tangential to a free surface, which corresponds to the kinematic condition, *v·n* = 0. This condition requires the starting line of the free surface as initial condition. The starting line of the free surface is the intersection of nozzle wall and surface of Extrudate in this study. Lastly, the boundary condition at the flow outlet (the end of extrudate) is zero normal and tangential forces because it is assumed that a uniform velocity profile is reached at the end of the extrudate where no external stress is applied to the extrudate. Whole nozzle and extrudate was simulated without generating symmetry surfaces. Coordinate system is defined as following. Y axis is at the center of nozzles. Negative Y direction is extrusion direction. X axis and Z axis are perpendicular to Y axis. The center of coordinate system is located at the center of the nozzle exit where X = Y = Z = 0 for two types of nozzles. While coupled model is with fluid and filler, decoupled model, a model without the filler is applied in order to simplify the simulation process.

Velocity profile images of the fluid (Fig. 2a,b) clearly show higher velocity (red color) at the center of both nozzles. 10 equally spaced lines were defined on XY planes to analyze fluid dynamics more in detail and understand the fiber orientation. In case of the circular nozzle, 10 yellow vertical lines were defined on XY plane which lies on the axis of rotation (a black vertical line) of cylinder shape model of fluid in the nozzle as in Fig. 2a. Because of the symmetric structure of a circular nozzle, 10 lines were placed only on the half of the plane where X is positive. In case of a flat nozzle, 10 equally spaced lines were placed on XY plane which lies on the axis of rotation of cylinder shape part of fluid model and cuts the flat part through the largest diameter of the ellipse-like cross section. Because of the symmetric structure of a flat nozzle, 10 lines were placed only on the half of the plane where X is positive. Fluid velocities were calculated along these 10 lines first. Then the average velocity graphs in Fig. 2c–e were attained from these velocity graphs along 10 lines. First of all, equally spaced 10 lines were defined to obtain detailed local velocity data as the velocity profile is not uniform. Then, the average flow velocity was calculated from obtained data along equally spaced 10 lines in order to compare the major difference of fluid flow in two types of nozzles.

The fiber orientation is estimated by calculation of the second order orientation tensor, *A*_33_ by using velocity gradients of fluid. The second order orientation tensor is defined as ${A}_{ij}={\oint }^{}{p}_{i}{p}_{j}{\psi }({\boldsymbol{p}})d{\boldsymbol{p}}$ . Here, ***p*** is the unit vector in the direction of primary axis of the fiber. *p*_1_ = sinθ cosφ, *p*_2_ = sinθ sinφ, *p*_3_ = cosθ, θ and φ are angles between coordinate axis and vector ***p***. The *A*_33_ component shows the fiber alignment according to the extrusion direction^21^. The velocities and velocity gradients can be calculated along 10 equally spaced lines from CFD results. If *A*_33_ is close to 1, it means a nearly uniaxial alignment of fibers along the extrusion direction. After the nozzle exit, *A*_33_ decreases mainly due to the expansion flow and the negative elongation component^21^. In this simulation, calculated *A*_33_ component varies from 0.94 to 1.00 for a flat nozzle and from 0.97 to 1.00 for a circular nozzle along 10 equally spaced lines around nozzle exits. The difference in calculated *A*_33_ component is not so enormous, but the fact that *A*_33_ component shows higher value in case of a circular nozzle reflects that there is more aligned distribution of fillers in case of a circular nozzle. In addition to that, velocity Y, velocity Z, and velocity X represents different extrusion for a circular and a flat nozzles. The aligned and random distribution of fillers depending on nozzle shapes can be understood by investigation of fluid movement. The fluid velocity graphs along equally spaced lines explain how fluid moves. The focus is especially on the nozzle exit because the movement of fluid at the nozzle exit will determine the orientation of fillers in printed samples.

After the nozzle exit (Y = 0), for the flat nozzle, filler alignment in Y direction is decreased as Y value is decreased due to two major changes. First, velocity Y (on average) decreases as the Y is decreased from positive to negative value. (The flat nozzle case shows steeper decreasing slope compared to the circular nozzle case). At the nozzle exit, the average of velocity Y of flat nozzle case is about 1.3 times higher than the average velocity Y of circular case (7.7 & 5.8) Second, the absolute value of velocity Z and velocity X show the highest value near the nozzle exit. Fillers will be more likely moving along Z and X axis direction (Expansion flow)^21^ instead of aligning along extrusion direction.

On the other hand, a circular nozzle case shows lower absolute value of average velocity Z and average velocity X compared to flat nozzle at the nozzle exit. At the nozzle exit, the average of velocity Z of flat nozzle is about 17.1 times faster than the average of velocity Z of circular nozzle (1.025 & 0.06). The average of velocity X of flat nozzle is about 1.8 times faster than the average velocity X of circular nozzle (7.7 & 4.2). Over all, velocity graphs along 10 lines of the flat nozzle shows larger deviation (the difference between values of each graph from the average graph) compared to velocity graphs along 10 lines of the circular nozzle. This also explains the reason why flat nozzle samples show random distribution of AgNWs while the circular nozzle samples show aligned distribution of AgNWs.

**Figure 3:**
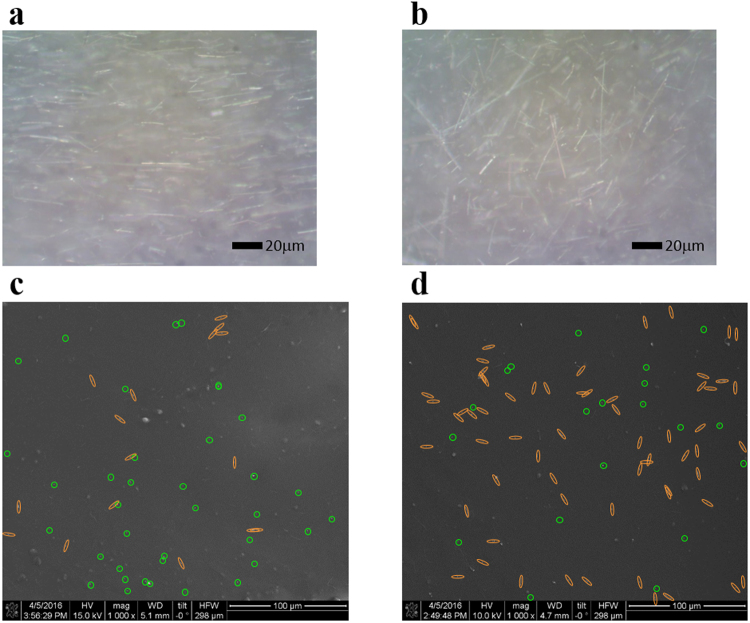
Filler distribution morphology. Optical microscope images of Print & Cure samples printed through a circular nozzle **(a)** and a flat nozzle **(b)** and SEM images of cross sections of samples printed through a circular nozzle **(c)** and a flat nozzle **(d)**.

Microscopic images in Fig. 3a,b show aligned and random distribution of AgNWs in P&C resin matrix depending on nozzle shapes. The sample printed with a circular nozzle shows aligned distribution of AgNWs (Fig. 3a). The sample printed with a flat nozzle shows random distribution of AgNWs (Fig. 3b). This was observed similarly in many areas of each sample. And this matches with the simulation result.

SEM images of the cross section of samples at in Fig. 3c,d show the difference between random and aligned AgNW orientation. In the aligned AgNWs sample printed with a circular nozzle, the cross section is showing many dots and smaller number of lines. This happens because AgNWs are aligned along the printing direction, and the cross section is perpendicular to this printing direction. AgNWs aligned to the printing direction will show dot images in cross section, while AgNWs randomly distributed will show line images. Only clear white dots or lines were counted, blurry larger dots or thicker lines were not counted. Thus, majority of AgNWs (~75%) are showing their cross section as dots at the cross section of the sample printed from a circular nozzle. On the other hand, the sample printed with a flat nozzle shows many lines (~75%) and smaller number of dots (~25%) because majority of AgNWs are lying with different angles at the cross section of the sample as a random distribution and are showing their surface of side as lines.

**Figure 4:**
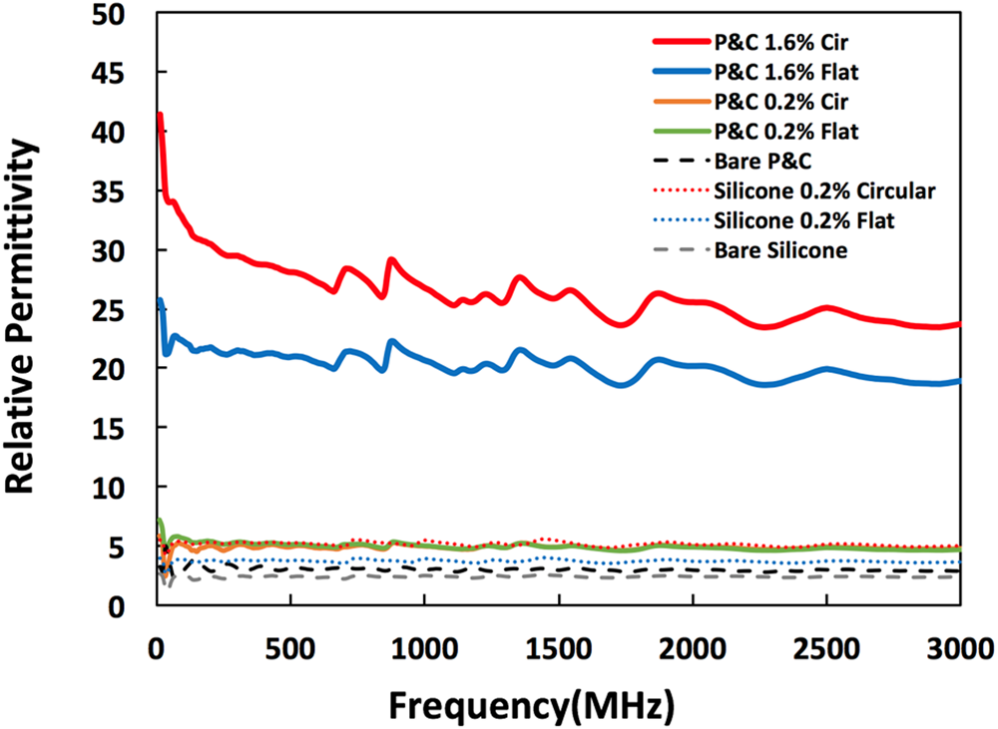
Permittivity of Print & Cure (AgNW vol. fraction: 0, 0.2, 1.6%) and Silicone (AgNW vol. fraction: 0, 0.2%) samples printed with a circular or a flat nozzle.

Dielectric permittivity was measured by R&S® ZND Vector Network Analyzer (VNA), connected with DAK-12 dielectric measurement system. Figure 4 compares the compensated permittivity which is the measured permittivity divided by the weight of each sample in order to compensate the difference of thickness of each sample. The area of each sample is the same. The thickness of a sample is proportional to the volume of a sample. Thus, we assumed there is a linear relationship between the thickness of samples and permittivity as the linear relationship between AgNW volume fraction and permittivity^26^. The compensated permittivity of each samples was obtained by calculating permittivity of 1 gram sample using measured permittivity, sample weight, and the permittivity of perfect vacuum which is 1 as shown in the following equation (2).

2 $${\rm{Compensated}}\,{\rm{permittivity}}=({\rm{Measured}}\,{\rm{permittivity}}-1)/({\rm{sample}}\,{\rm{weight}})+1$$

where, measured permittivity is a relative permittivity (ε_r_ = ε/ε_0_).

For P&C 0.2% Flat and P&C 0.2% Circular (Cir) cases, there is not so much difference in permittivity. However, P&C 1.6% Cir case has about 30% higher permittivity compared to P&C 1.6% Flat case. This is because Cir has more alignment of AgNWs. These aligned AgNWs will make a lot of couples of two parallel AgNWs which will work like micro capacitors. The permittivity is the amount of charge required to apply 1 unit of electric flux in a medium. Higher dielectric permittivity means higher performance to resist an applied electric field and enables storing more electrical energy in a limited volume, which will be useful for energy storage devices^3^.

**Figure 5:**
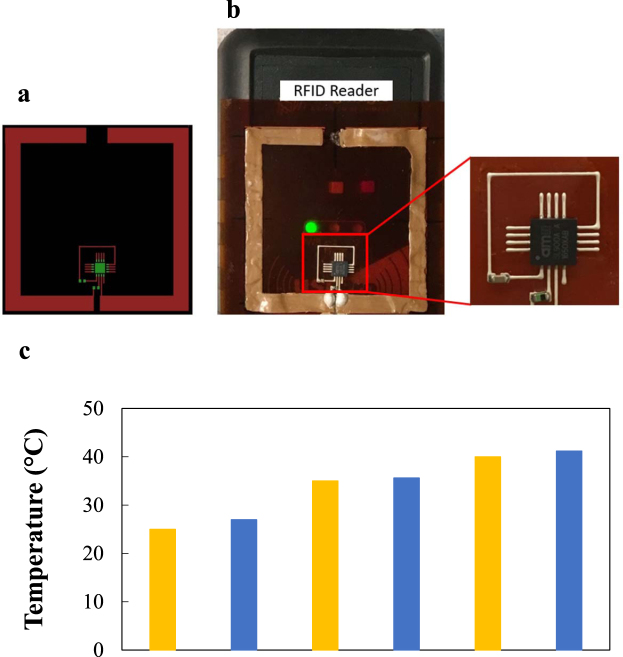
The RFID tag design and the detection of a fabricated RFID tag by an RFID reader. **(a)** Design of antenna and circuit layout **(b)** Detection of the RFID tag consisted of an antenna printed with AgNW (1.5% vol. fraction) and P&C resin composite through a flat nozzle and interconnected with an RFID chip **(c)** Temperature sensing result from 25 to 40 °C: yellow bars show heater’s temperature (25, 35, and 40 °C) and, blue bars show corresponding temperature detected by the RFID tag (26, 35, and 41 °C).

As a demonstration, a passive radio frequency identification (RFID) tag with temperature sensibility was designed to have a circuit and an antenna with a resonant frequency as 910 MHz which is compatible with the RFID reader (Fig. 5a). This RFID Antenna was printed by P&C system on a piece of 127 µm Polyimide (PI) film, Kapton® HPP film as shown in Fig. 5b. The antenna was printed with the P&C resin with 1.5 vol. % AgNWs through a flat nozzle so that the randomly distributed AgNWs in printed antenna form a conductive network. This antenna was put into an oven for annealing AgNWs at 200 °C for 10 minutes after printing to attain enhanced conductivity. The circuit part of the RFID tag was manufactured by printing an interconnection and soldering components on it. The interconnection was printed by a Printed Circuit Board (PCB) printer, Voltera V1, with an appropriate dimension for 0603 footprint electronic components and an RFID chip because the resolution of V1 printer met the printing requirement. SL900A as an RFID chip, 47 nH SMD inductor, and 2.2 µF SMD capacitor were soldered on the interconnection. The inductor placed between the RFID chip and antenna makes a large reading range, and the capacitor makes the signal smooth. This RFID tag was well identified by the RFID reader which is compatible with the North American UHF RFID band (902–928 MHz) within the distance up to 1 cm. And the temperature sensing information in the RFID chip was read successfully by the reader at three different temperature levels (Fig. 5c).

## Conclusion

In this report, photo-curable 3D printing system has been designed to optimize the printing of a photo-curable nanocomposites composed of photo-curable resin and AgNWs. Specially, the permittivity difference has been observed from 3D printed samples with same concentration of AgNWs when they are 3D printed with different nozzles. The computational study confirms that the extrusion printing with a circular nozzle generates an aligned distribution of fillers, and the extrusion printing with a flat nozzle induces a random distribution of fillers. Utilizing the conductively percolated network realized by printing through a flat nozzle, an RFID antenna is fabricated. The antenna designed to have a resonant frequency as 910 MHz was printed on polyimide substrate for the demonstration of RFID communication and temperature sensing. Therefore, the study on the control of filler orientation in prints ushers the way to control composite material’s electrical properties by nozzle shape dependent 3D printing. This can open a new platform of extrusion printing for controlling material’s property by design of 3D printing parameters. The effect of nozzle diameters on overall properties will be investigated in future publications by additional experiment and parametric simulation. We expect that nozzle’s larger diameter will generate enhanced alignment of AgNWs in case of a circular nozzle and more random distribution of AgNWs with a flat nozzle compared to smaller diameter nozzles because a larger diameter nozzle will allow more variation of local velocity across the cross section of the nozzle compared to a smaller diameter nozzle.

## Materials and Methods

### Photo-curable extrusion printing system - Print & Cure

4 Laser diodes were mounted right beside the position of the printing nozzle in the printing head of extrusion printing system to conduct photo-curable extrusion printing. The wavelength of UV light of a diode is 405 nm, and the optical power of a diode is 120 mW. To supply a stable 5 V DC voltage for each diode, drivers for diodes were made with a regulator 7805, two capacitors of 0.33 μF and 10 μF, and a resister of 47 Ω. The power was supplied from the RamBo board of a delta FDM 3D printer (Orion, SeeMeCNC). A paste extrusion system (Discov3ry, Structur3d Printing) was modified to save printing material. Two syringes were connected by a tube which is filled with water in order to transfer the pressure from the primary syringe to the syringe at the extrusion side. The motor of Discov3ry extruder gives pressure to the primary syringe, and the pressure is transferred to the smaller syringe at the extrusion side.

### Preparation of AgNW/P&C resin printed samples

AgNWs were prepared by polyol process which make AgNWs with high aspect ratio. The aspect ratio is about 200. The diameter and length of AgNWs are 100 nm and 20 μm respectively. AgNWs are mixed with P&C resin which is made of an oligomer (Urethane triacrylate), a photo initiator (Phenylbis phosphine oxide), and a monomer (Methacrylic acid). Prepared composite material is mixed thoroughly by a vortex mixer and a magnetic stirrer. To study permittivity of AgNW/P&C resin, 4 samples are printed with two different nozzles and two different AgNW vol. fractions of 0.2% and 1.6%. Assuming that the percolation threshold of AgNWs is around 0.7% volume fraction based on our group’s previous research with same AgNWs^2^, Concentrations of 0%, 0.2%, and 1.6% volume fraction were chosen in order to compare three different cases such as pristine polymer matrix, PNC with AgNWs of volume fraction under the percolation threshold, and PNC with AgNWs of volume fraction over the percolation threshold.

### Simulation of printing composite

Fluid models of composite material in a circular and a flat nozzle tips and extrudates are generated with the Soildworks. After fluid models are made, these geometry files are saved as parasolid format (.x_t). By using these parasolid files, meshes were created in a simulation software, Ansys Workbench (ANSYS, Inc.). After improving mesh quality by CFD (Computational Fluid Dynamics), models are analyzed through polyflow extrusion of the Ansys Workbench to find velocity profile of composite material. According to the mesh quality report, number of total elements of the circular nozzle case is 204222. Among these elements, 96% have quality value greater than 0.8227535. On the other hand, the flat nozzle case has a larger number of total elements, 666816 compared to the circular nozzle case. It’s mainly because mesh elements in the shape transition part of the flat nozzle (from cylindrical to flat) should be refined enough to meet the convergence criteria. Among these elements, 86.3% have quality value greater than 0.8177935. 77.0% of elements have skew value less than 0.9295337 which is acceptable.

## References

[1] Zhang, X. *et al.* Magnetoresistive Conductive Polyaniline–Barium Titanate Nanocomposites with Negative Permittivity. *J. Phys. Chem. C* **116**, 15731–15740 (2012).

[2] Park JS, Kim T, Kim WS (2017). Conductive Cellulose Composites with Low Percolation Threshold for 3D Printed Electronics. Sci. Rep..

[3] Gao, J. *et al*. Designing High Dielectric Permittivity Material in Barium Titanate. *J. Phys. Chem. C* **121**, 13106–13113 (2017).

[4] Prateek TVK, Gupta RK (2016). Recent Progress on Ferroelectric Polymer-Based Nanocomposites for High Energy Density Capacitors: Synthesis, Dielectric Properties, and Future Aspects. Chem. Rev..

[5] Wang G, Huang Y, Wang Y, Jiang P, Huang X (2017). Substantial enhancement of energy storage capability in polymer nanocomposites by encapsulation of BaTiO3 NWs with variable shell thickness. Phys. Chem. Chem. Phys..

[6] Patton ST (2016). Multiphysics characterization of multi-walled carbon nanotube thermoplastic polyurethane polymer nanocomposites during compression. Carbon.

[7] Kim P (2007). Phosphonic Acid-Modified Barium Titanate Polymer Nanocomposites with High Permittivity and Dielectric Strength. Adv. Mater..

[8] Zhang X, He Q, Gu H, Wei S, Guo Z (2013). Polyaniline stabilized barium titanate nanoparticles reinforced epoxy nanocomposites with high dielectric permittivity and reduced flammability. J. Mater. Chem. C.

[9] Madusanka N (2016). Nanocomposites of TiO2/cyanoethylated cellulose with ultra high dielectric constants. Nanotech..

[10] Kumar GS, Vishnupriya D, Chary KS, Patro TU (2016). High dielectric permittivity and improved mechanical and thermal properties of poly(vinylidene fluoride) composites with low carbon nanotube content: effect of composite processing on phase behavior and dielectric properties. Nanotech..

[11] Zeraati AS, Arjmand M, Sundararaj U (2017). Correction to “Silver Nanowire/MnO2 Nanowire Hybrid Polymer Nanocomposites: Materials with High Dielectric Permittivity and Low Dielectric Loss”. ACS Appl. Mater. & Inter..

[12] Arbatti M (2007). Ceramic–Polymer Composites with High Dielectric Constant. Adv. Mater..

[13] Hu N, Karube Y, Yan C, Masuda Z, Fukunaga H (2008). Tunneling effect in a polymer/carbon nanotube nanocomposite strain sensor. Acta Mater..

[14] Sun Y, Mayers B, Herricks T, Xia Y (2003). Polyol Synthesis of Uniform Silver Nanowires: A Plausible Growth Mechanism and the Supporting Evidence. Nano Lett..

[15] Arjmand M, Moud AA, Li Y, Sundararaj U (2015). Outstanding electromagnetic interference shielding of silver nanowires: comparison with carbon nanotubes. RSC Adv..

[16] Compton BG, Lewis JA (2014). 3D Printing: 3D-Printing of Lightweight Cellular Composites (Adv. Mater. 34/2014). Adv. Mater..

[17] Tjong SC (2011). Polymer nanocomposite bipolar plates reinforced with carbon nanotubes and graphite nanosheets. Ener. & Env. Sci..

[18] Martin-Gallego M, Lopez-Manchado MA, Calza P, Roppolo I, Sangermano M (2014). Gold-functionalized graphene as conductive filler in UV-curable epoxy resin. J. Mater. Sci..

[19] Du F, Fischer JE, Winey KI (2005). Effect of nanotube alignment on percolation conductivity in carbon nanotube/polymer composites. Phys. Rev. B.

[20] Tang H, Malakooti MH, Sodano HA (2013). Relationship between orientation factor of lead zirconate titanate nanowires and dielectric permittivity of nanocomposites. Appl. Phys. Lett..

[21] Heller BP, Smith DE, Jack DA (2016). Effects of extrudate swell and nozzle geometry on fiber orientation in Fused Filament Fabrication nozzle flow. Add. Man..

[22] Lebel LL, Aissa B, Khakani MAE, Therriault D (2010). Ultraviolet-Assisted Direct-Write Fabrication of Carbon Nanotube/Polymer Nanocomposite Microcoils. Adv. Mater..

[23] Cooperstein I, Layani M, Magdassi S (2015). 3D printing of porous structures by UV-curable O/W emulsion for fabrication of conductive objects. J. Mater. Chem. C.

[24] Verweyst BE, Tucker CL (2002). Fiber Suspensions in Complex Geometries: Flow/Orientation Coupling. The Canadian J. Chem. Eng..

[25] Cross MM (1965). Rheology of non-Newtonian fluids: A new flow equation for pseudoplastic systems. J. Coll. Sci..

[26] Mi H-Y, Li Z, Turng L-S, Sun Y, Gong S (2014). Silver nanowire/thermoplastic polyurethane elastomer nanocomposites: Thermal, mechanical, and dielectric properties. Mater. & Des..

